# Effectiveness of total hip arthroplasty versus non-surgery on patient-reported hip function at 3 months: a target trial emulation study of patients with osteoarthritis

**DOI:** 10.2340/17453674.2025.43332

**Published:** 2025-04-14

**Authors:** Amanda D KLAASSEN, Wiard JORRITSMA, Nienke W WILLIGENBURG, Carina L E GERRITSMA, Bas L E F Ten HAVE, Dirk Jan F MOOJEN, Maaike G J GADEMAN, Rolf H H GROENWOLD, Rudolf W POOLMAN

**Affiliations:** 1 Department of Orthopedic Surgery, Joint Research, OLVG Hospital, Amsterdam; 2 Department of Orthopedics, Leiden University Medical Center, Leiden; 3 Santeon Better Together Value-Based Health Care (VBHC) Program, Utrecht; 4 Department of Quality and Improvement, OLVG Hospital, Amsterdam; 5 Department of Orthopedic Surgery, Martini Hospital, Groningen; 6 Department of Clinical Epidemiology, Leiden University Medical Center, Leiden, the Netherlands

## Abstract

**Background and purpose:**

This study introduces an innovative research design in the field of orthopedics, using a target trial emulation approach. We aimed to assess the causal effects of total hip arthroplasty (THA) compared with nonoperative treatment in reducing patient-reported hip disability at 3 months in patients with osteoarthritis, using real-world data.

**Methods:**

We emulated a target trial using real-world data of 2 Dutch hospitals between April 2020 and January 2022. Patients diagnosed with hip osteoarthritis and eligible for primary THA were included in the study. During the COVID-19 pandemic, THA was often cancelled due to external factors (i.e., limited operating room capacity, or surgeon unavailable due to quarantine rules), resulting in an arbitrary allocation of patients to THA (n = 132) or non-THA (n = 60). We compared changes in hip disability, measured using the Hip disability and Osteoarthritis Outcome Score Physical function Short form (HOOS-PS), between the THA group at 3 months postoperatively and the non-THA group at ≥3 months post waiting-list. Linear regression analysis, adjusting for potential confounders, was used to compare between-group differences.

**Results:**

THA showed preferable outcomes compared with non-THA, indicated by a difference of –33 points (95% confidence interval [CI] –37 to –28) on the HOOS-PS. Patients in the THA group demonstrated a clinically significant improvement in hip function, with a mean change of –27 points (CI –31 to –24), while the control group showed no improvement with a mean change of 7 points (CI 3–11) on the HOOS-PS.

**Conclusion:**

THA significantly improves hip function in osteoarthritis patients, surpassing the outcomes observed in the non-surgery group.

Total hip arthroplasty (THA) is one of the most frequently performed procedures in orthopedic surgery [1,2]. Observational studies and registry outcomes suggest that THA is an effective treatment to reduce pain and improve hip function and quality of life in patients with osteoarthritis of the hip [1,3]. Until recently, no studies were available demonstrating the efficacy of total hip arthroplasty (THA) evaluated through a randomized controlled trial (RCT) [4]. The well-known clinical benefits of THA may lead to difficulties in patient inclusion, especially among those with severe osteoarthritis (OA). The perceived lack of clinical equipoise between THA and nonoperative strategies such as resistance training could make patients with severe OA and debilitating pain unwilling to participate in a study where there is a chance to be randomized to a non-surgical treatment option. Similarly, surgeons may be uncomfortable recruiting patients with severe symptoms, potentially denying the patient a surgical option known to be effective. Consequently, results of an RCT may have limited generalizability. Target trial emulation is a framework that aims to mimic an ideal study (in this case, an RCT between surgery and non-surgery) using real-world data [5,6].

During the COVID-19 pandemic, elective orthopedic interventions were defined as not urgent and, therefore, often cancelled. The National Institute for Public Health and the Environment (RIVM) estimated that in 2020 and 2021 in total an estimated 32,000 quality-adjusted life years (QALYs) were not realized due to postponing THA in patients with osteoarthritis of the hip in the Netherlands [7]. Enhanced comprehension of THA’s treatment effect relative to non-surgical options could aid in better prioritizing surgical interventions.

In this study, we used target trial emulation to assess the effect of THA at 3 months postoperatively, compared with non-THA (i.e., cancelled surgery). Primary objective was to measure disability with the Hip disability and Osteoarthritis Outcome Score Physical function Short form (HOOS-PS) in patients with end-stage hip osteoarthritis. Secondary objectives were to validate the assumption of comparability between the THA and the non-surgery group and to assess the effect of THA on pain. We hypothesize that THA is effective compared with non-surgery in reducing hip disability.

## Methods

### Study design: target trial specification

We specified a target trial protocol to investigate the effect of THA surgery on hip disability and pain compared with no surgery in patients with hip osteoarthritis eligible for THA. While a formal guideline on reporting of a target trial and its emulation is currently being developed, we have relied on the STROBE guideline and the latest literature to ensure comprehensive reporting of our target trial and its emulation [8,9]. In this study, we used a dataset of routinely collected health information to emulate a target trial that compares patients who underwent THA surgery with patients who did not receive THA and remained on the waiting list. An overview of eligibility criteria and other protocol elements of the target trial specification and the target trial emulation are presented in Table 1.

**Table 1:** Description of target trial specification and target trial emulation

Target trial specification	Target trial emulation
**Eligibility criteria**	
Age ≥ 18 Hip osteoarthritis Indication THA Intake questionnaire completed	Similar to target trial and additionally Intake questionnaire completed between April 2020 and January 2022 Intake questionnaire completed ≤ 100 days before THA indication Exclusion: THA on both sides between April 2020 and January 2022
**Treatment strategies**	
Elective primary THA Non-THA	Similar to target trial In group B, surgery was cancelled due to COVID and patients remained on the waiting list
**Assignment procedures**	
Random group assignment: (A) THA and (B) non-THA	Pseudo-random group assignment and adjustment for confounding factors at baseline: (A) THA and (B) non-THA (i.e., patients who were still on the waiting list within 180 days after intake)
**Outcomes**	
Hip function (HOOS-PS) Hip pain (NRS) Improvement function (anchor-based) Improvement pain (anchor-based)	Same as in the target trial
**Follow-up questionnaire**	
3 months after baseline (i.e., 3 months after randomization)	THA group (A): Sent at 3 months postoperatively. Follow-up time from intake can vary, as patients were on the waiting list before surgery. Non-THA group (B): At least 3 months after intake.Follow-up time from intake may vary, as patients were on the waiting list before planned surgery and follow-up questionnaires were sent in batches. Patients who completed the follow-up questionnaire after April 2022 were excluded from analysis.
**Causal contrast(s)**	
Main analysis: Per protocol effect	Observational data, per protocol effect
**Analysis plan**	
Comparison between treatment groups, estimated using linear regression analysis, adjusted for baseline scores. Comparison between treatment groups, estimated using linear regression, adjusted for baseline scores and age, sex, PT before baseline, and hospital	Logistic regression analysis to check the assumption of independence between patient characteristics and received treatment. Comparison between treatment groups, estimated using linear regression, adjusted for baseline scores and age, sex, PT before baseline, and hospital. Sensitivity analysis on imputed data to address missing data.
**Specification of time zero (i.e., baseline)**	
Date of randomization	Baseline is the date of completing the intake questionnaire

THA = total hip arthroplasty; HOOS-PS = Hip disability and Osteoarthritis Outcome Score-Physical function Short form; NRS = numerical rating scale; PT = physical therapy.

### Outcomes

Clinical outcomes on 3 domains as outlined by the OMERACT-OARSI Core Domain Set were measured using validated patient-reported outcome measures (PROMs): physical function using HOOS-PS ranging from 0 to 100 with lower scores reflecting better physical function, pain during weightbearing using the numerical rating scale (NRS), and the patient’s global assessment of the target joint using an anchor-based question [10]. The anchor-based question was administered for improvement in hip function and hip pain, consisting of a 7-item Likert scale from “very much improved” as the best option available and “very much deteriorated” as the worst option available.

### Target trial emulation

To investigate the effect of THA surgery on hip disability and pain, we emulated the target trial using observational real-world data of 2 Dutch hospitals participating in the Santeon Better Together value-based health care (VBHC) program during the COVID-19 pandemic. During the pandemic, primary THA surgery was considered a non-urgent intervention, regardless of the severity of symptoms and patient characteristics. Therefore, THA was often cancelled due to external factors, e.g., a changed government or hospital policy effective the next day or a surgeon having to quarantine due to (a relative with) a COVID infection. These issues resulted in a pseudo-random allocation of patients to either surgery or non-surgery, beyond the control of patients, clinicians, and researchers, on whether a patient could undergo the planned THA. Additionally, in the emulated trial, we adjusted for potential confounding factors measured at baseline to ensure comparability between the 2 groups.

### Population

Patients diagnosed with end-stage hip osteoarthritis were eligible for the study when they fully completed a preoperative intake questionnaire between April 2020 and January 2022, within 100 days before being placed on the THA waiting list. Group assignment was based on whether surgery could be performed, or whether planned surgery was cancelled or could not be planned, leaving patients on the waiting list. The THA group consists of patients who received surgery within 180 days after the intake questionnaire was completed and the control group consists of patients who did not receive surgery within 180 days after the intake questionnaire was completed. This cutoff point was based on the regular waiting time for THA surgery in both hospitals, which was approximately 3 to 6 months.

During the COVID-19 pandemic, an additional questionnaire was administered temporarily by the department of quality and improvement for orthopedic surgery, to monitor the health status of patients on the waiting list in 2 Dutch hospitals (OLVG, Amsterdam and Martini, Groningen). Since this questionnaire was not integrated in standard care, sending out the questionnaire to the control group was stopped for unknown reasons and not all patients in the control group were invited to complete the follow-up questionnaire. To address missing outcome data, the un-collected responses were imputed and analyzed in a sensitivity analysis, which has been described in detail under “sensitivity analysis.” Follow-up questionnaires were sent online to patients at 3 months postoperatively (n = 146) and to those who were on the waiting list (n = 64) for THA for at least 3 months. No crossover between groups occurred because all THA patients completed the 3-months questionnaire postoperatively and all non-THA patients completed the follow-up questionnaire while they were still on the waiting list. For example, a non-THA patient who would be scheduled for THA on day 200 would be included in the non-THA group with a completed non-THA questionnaire prior to surgery. Follow-up time in days was measured between the date of the intake questionnaire and the follow-up questionnaire. Given that patients were on the waiting list before (planned) surgery, follow-up time from intake varied and could exceed 3 months. Follow-up questionnaires were included for analysis when completed before April 2022. Patients who underwent bilateral THA between April 2020 and January 2022 were excluded.

### Statistics

First, the assumption of comparability between groups was assessed, using a logistic regression analysis. Group (THA/non-THA) was included as dependent variable, and age (continuous), sex (M/F), HOOS-PS at baseline (continuous), pain at baseline (NRS, continuous), physical therapy before the hospital visit (yes/no), and hospital (hospital 1/hospital 2) were added to the model as independent variables. Odds ratios were reported with 95% confidence intervals (CI) and were considered significant if their CI excluded 1.

In our main analysis, we compared the change in hip disability between the 2 groups using a multivariable linear regression analysis, where the HOOS-PS score postoperatively was the dependent variable, group (THA/non-THA) was added as the primary independent value of interest and age, sex (M/F), HOOS-PS score at baseline, NRS pain score at baseline, physical therapy before baseline (yes/no), and hospital (hospital 1/hospital 2) were added as independent variables to correct for potential confounding. As a secondary analysis, linear regression analyses were performed to assess the effect of THA on the change in hip pain and on the anchor questions for hip function and pain, while adjusting for the same independent variables as described above. Between-group effects were presented with CIs. Statistical analyses were performed using R version 4.4.1 (R Foundation for Statistical Computing, Vienna, Austria). A difference was considered clinically relevant when exceeding the minimal clinically important difference (MCID) of 23 points for the HOOS-PS [11].

### Sensitivity analysis

To assess the robustness of our findings, we conducted a sensitivity analysis on the dataset containing all allocated patients, incorporating imputed values for missing outcome data. Missing data were addressed using the Multiple Imputation by Chained Equations (MICE) method [12]. Predictive mean matching was performed, using 100 iterations, generating 50 datasets. The imputation model included the following variables: group, age, sex, HOOS-PS baseline, NRS-baseline, physical therapy, hospital, follow-up HOOS-PS, follow-up NRS, and anchor-based questions. The main analysis outlined in the statistical methods section was repeated on the imputed datasets and results were pooled using Rubin’s rule. Results of linear regression analysis are presented in the supplementary data for unadjusted results, adjusting solely for baseline scores, and adjusted results, accounting for all potentially confounding variables.

### Ethics, registration, use of AI tools, funding, and disclosures

Ethical approval for this study was obtained from the local review committees of the participating hospitals (registration number: SDB 2023-002) and conducted according to the Declaration of Helsinki (2013). Results are presented according to the STROBE guidelines. The study protocol is registered at ClinicalTrials.gov (NCT06263569). Data in this retrospective study was completely anonymized before processing and analyzing. ChatGPT was used to optimize the flow and clarity of already written textual content. No funding was received for this study. The authors declare that they have no conflicts of interest regarding this research. Complete disclosure of interest forms according to ICMJE are available on the article page, doi: 10.2340/17453674.2025.43332

## Results

### Study population and follow-up

**Figure 1:**
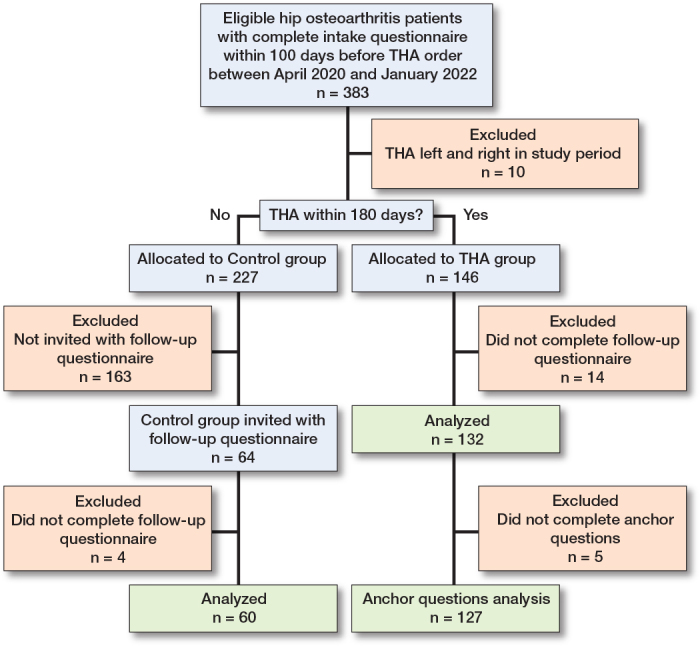
Patient flow chart. THA = total hiparthroplasty.

**Figure 2:**
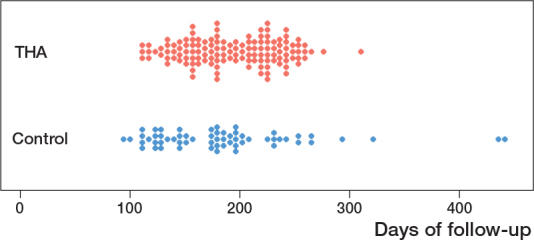
Follow-up time (number of days between intake and follow-up questionnaire) for individual patients in the 2 groups.

383 patients with hip osteoarthritis, who completed the intake questionnaire within 100 days before the THA order, were deemed eligible (Figure 1). Of these, 10 patients were excluded due to undergoing bilateral THA within the study period. The remaining patients were divided into 2 groups: the THA group (n = 146) and the control group (n = 227). In the THA group, 14 patients did not complete the follow-up questionnaire, resulting in 132 patients who were included in the analysis. Additionally, 5 patients failed to complete the anchor question and were excluded from the analysis on the anchor question. Patients in the control group remained on the waiting list, due to unexpected surgery cancellations or logistical restrictions that prevented surgery planning during the COVID-19 pandemic. Of these, 64 patients were invited to complete a follow-up questionnaire, with 60 patients ultimately included in the analysis. The remaining 163 patients in the control group were not invited, as the follow-up questionnaire was only administered temporarily in both hospitals. Demographics and patient reported outcomes for both groups are displayed in Table 2. Follow-up times for individual patients are shown in Figure 2 (see Appendix).

**Table 2:** Baseline characteristics and follow-up time for patients in the THA and non-THA group. Values are presented as median (IQR) or n (%)

Factor	Complete cases dataset		Imputed dataset	
	THA	Non-THA	THA	Non-THA
	(n = 132)	(n = 60)	(n = 146)	(n = 227)
Age	71 (65–76)	69 (63–75)	71 (65–77)	69 (63–74)
Male sex	32 (24)	25 (42)	36 (25)	97 (43)
HOOS-PS baseline	46 (34–61)	38 (30–51)	46 (34–61)	46 (34–56)
NRS pain baseline	8 (7–9)	8 (6–8)	8 (7–9)	8 (7–8)
PT preoperative	83 (63)	38 (63)	91 (62)	148 (65)
Hospital 1	43 (33)	36 (60)	93 (64)	92 (41)
Follow-up, days	194	179	–	–
(IQR)	(157–226)	(133–204)		

For abbreviations, see Table 1; IQR = interquartile range.

### Validation of assumption

The assumption of comparability between groups seemed appropriate for most characteristics, except for the variable “Hospital”. Patients who visited Martini Hospital had a higher probability of being allocated to the THA group (OR 3.1, CI 1.6–6.2). All odds ratios and CI are presented in Table 3 (see Appendix).

**Table 3:** Logistic regression analysis to check the assumption of comparability between groups at baseline: variables with a potential effect on group allocation (THA vs non-THA)

Variable	Odds ratio (CI)
Age	1.02 (0.98–1.05)
Male sex	0.55 (0.28–1.11)
HOOS-PS at baseline	1.01 (0.99–1.03)
NRS pain at baseline	1.20 (0.98–1.48)
Followed physical therapy	1.04 (0.51–2.07)
Hospital	3.12 (1.61–6.18)

For abbreviations, see Table 1; CI = 95% confidence interval.

### Outcomes

In the THA group, patients showed clinically relevant improvement in hip function and pain with a mean change score of –27 (CI –31 to –24) points on the HOOS-PS and –5.3 (CI –5.8 to –4.8) points on the NRS pain during weightbearing (Table 4). In the control group, however, patients did not show improvement with a mean change score of 7.2 (CI 3.2–11) points on the HOOS-PS and 0.0 (CI –0.6 to 0.6) points on the NRS. Mean improvement on the anchor questions for hip function and pain were 6.0 (CI 5.8–6.2) and 6.2 (CI 6.0–6.4) respectively, in the THA group. The non-THA group reported mean improvement on the anchor questions for hip function and pain of 4.1 (CI 3.7–4.5) and 4.0 (CI 3.6–4.5) points, respectively. A score of 6 represents the category ‘much improved’, while a score of 4 represents the category ‘unchanged’.

**Table 4:** Mean (CI) outcomes for patient-reported hip function, pain, and anchor questions at follow-up and adjusted between group effects of the THA group compared with the control group

Outcome	THA group		Non-THA group		Between-group difference (CI)
	n	mean (CI)	n	mean (CI)	
Hip function					
HOOS-PS	132	20 (18–23)	60	50 (45–54)	–33 (–37 to –28)
Anchor function	127	6.0 (5.8–6.2)	60	4.1 (3.7–4.5)	2.0 (1.6 to 2.5)
Hip pain					
NRS pain	132	2.4 (1.9–2.8)	60	7.0 (6.3–7.6)	–4.8 (–5.6 to –3.9)
Anchor pain	127	6.2 (6.0–6.4)	60	4.0 (3.6–4.5)	2.4 (2.0 to 2.8)

Between-group effects were obtained using linear regression analysis, adjusted for age, sex, baseline HOOS-PS score, baseline NRS pain score, physical therapy, and hospital.

For abbreviations, see Table 1; CI = 95% confidence interval.

The primary analysis showed a group effect of –33 (CI –37 to –28; P < 0.001) points on the HOOS-PS in favor of the THA group compared with the control group (Table 4). Regression analysis for secondary outcomes showed between group effects of –4.8 (CI –5.6 to –3.9; P < 0.001) points on the NRS for pain; 2.0 (CI 1.6–2.5) points on the anchor question for hip function and 2.4 (CI 2.0–2.8) points on the anchor question for hip pain, in favor of the THA group.

### Sensitivity analysis

A sensitivity analysis was performed on the imputed data. Baseline characteristics for the “imputed” groups (i.e. including patients with missing data) show that most baseline characteristics were comparable to those observed for the “complete cases” groups (Table 2). Small differences were observed for the variable “Hospital”: in the “imputed” sample a higher proportion of THA patients and a lower proportion of non-THA patients were included in Hospital 1, compared with the “complete cases” dataset. Additionally, HOOS-PS scores in the non-THA group were slightly higher in the “imputed” sample than in the “complete cases” sample. Results of the sensitivity analysis were consistent with our primary analysis and showed similar and even slightly larger differences in favor of the THA group, with an adjusted between-group effect of –35 points (CI –40 to –30) on the HOOS-PS in favor of the THA group. For the secondary outcomes, the adjusted between-group effects were –5.0 points (CI –5.7 to –4.4) on the NRS pain; 2.4 points (CI 2.0–2.8) on the anchor function and 2.8 (2.3–3.3) on the anchor pain (Table 5, see Appendix).

**Table 5:** Unadjusted and adjusted between group differences and least-squares (LS) means for patient-reported hip function, pain and the anchor questions of the THA group compared with the control group

Outcome	Unadjusted results					Adjusted results				
	THA group		Non-THA group		Between group	THA group		Non-THA group		Between group
	n	LS mean (CI)	n	LS mean (CI)	difference (CI)	n	LS mean (CI)	n	LS mean (CI)	difference (CI)
Complete cases dataset										
HOOS-PS	132	20 (17–22)	60	51 (47–54)	–31 (–35 to –26)	132	18 (15–21)	60	50 (47–54)	–33 (–37 to –28)
Anchor function	127	6.0 (5.7–6.2)	60	4.1 (3.8–4.4)	1.9 (1.5 to 2.3)	127	6.1 (5.9–6.4)	60	4.1 (3.8–4.4)	2.0 (1.6 to 2.5)
NRS pain	132	2.3 (1.9–2.8)	60	7.1 (6.4–7.7)	–4.7 (–5.5 to –3.9)	132	2.2 (1.7–2.7)	60	7 (6.3–7.6)	–4.8 (–5.6 to –3.9)
Anchor pain	127	6.2 (6.0–6.4)	60	4.0 (3.7–4.4)	2.2 (1.8 to 2.6)	127	6.4 (6.2–6.7)	60	4 (3.7–4.4)	2.4 (2.0 to 2.8)
Imputed dataset										
HOOS-PS	146	20 (18–23)	227	53 (49–57)	–33 (–38 to –30)	146	18 (15–21)	227	53 (49–57)	–35 (–40 to –30)
Anchor function	146	5.9 (5.7–6.2)	227	3.7 (3.4–4.1)	2.2 (1.8 to 2.6)	146	6.1 (5.9–6.4)	227	3.7 (3.4–4.1)	2.4 (2.0 to 2.8)
NRS pain	146	2.4 (2.0–2.8)	227	7.4 (6.9–7.9)	–5.0 (–5.7 to –4.3)	146	2.2 (1.8–2.7)	227	7.3 (6.7–7.8)	–5.0 (–5.7 to –4.4)
Anchor pain	146	6.2 (5.9–6.5)	227	3.6 (3.2–4.0)	2.6 (2.1 to 3.0)	146	6.4 (6.2–6.7)	227	3.6 (3.3–4.0)	2.8 (2.3 to 3.3)

Results were obtained using linear regression. Analysis was performed separately for complete cases and on the imputed dataset. Unadjusted models included only baseline scores as a covariate, adjusted models accounted for age, sex, baseline HOOS-PS score, baseline NRS pain score, physical therapy and hospital.

For abbreviations, see Table 1; CI = 95% confidence interval.

## Discussion

This is the first study applying target trial emulation to evaluate the effectiveness of an orthopedic procedure.

We aimed to assess the causal effects of THA compared with nonoperative treatment in reducing patient-reported hip disability at 3 months in osteoarthritis patients, using real-world data. In this target trial emulation study, we found a substantial and clinically relevant effect of total hip arthroplasty on improvement of hip function, compared with nonoperative treatment. While our study identified substantial improvements within the THA group compared with the non-THA group, the nature of our study design necessitates a nuanced interpretation regarding causal effects.

The observed effect sizes in our investigation are similar to the effect sizes of THA shown in observational studies. Based on data from the Dutch Arthroplasty Register (LROI), a median change score was observed of 30 points on the HOOS-PS and 5.1 points on the NRS pain from baseline to 3 months postoperatively [13]. A study comparing outcomes between international registries observed mean improvement in HOOS-PS scores ranging from 29 to 35 points and a pooled change in HOOS-PS of 32 (CI 24–39) points [14].

Recently, Frydendal et al. published the first RCT comparing the effects of THA and progressive resistance training in hip osteoarthritis patients [15]. That study demonstrated significantly superior hip function in the THA group measured using the Oxford Hip Score, supporting our findings. However, enrollment was challenging due to patients’ strong preference for THA, with only 14% of eligible participants included, suggesting potential selection bias in the study population although baseline values did not differ from standard patients eligible for THA.

By conducting a meta-analysis that combines the results of our study RCTs, we can achieve a more comprehensive and precise estimation of the effect size. This approach would not only integrate findings from different research methodologies but also potentially reconcile any variations in outcomes, thereby offering a more robust and nuanced understanding of the efficacy of THA compared with alternative treatments in this patient population.

An important step of our study was to validate the assumption of comparability between groups at baseline. Logistic regression analysis showed that the distribution of patient characteristics between the 2 groups was mostly similar. However, patients in 1 hospital had a higher probability of being allocated to the THA group which could indicate some degree of confounding bias. To accurately estimate effect sizes of THA compared with non-THA, confounding adjustment was performed using multivariable regression analysis [9,16]. The adjusted, observed between-group differences were in favor of the THA group and substantially exceeded the MCID known from the literature, which is 23 points for the HOOS-PS [11].

### Strengths

A strength of this study is the target trial emulation design. A review by Blom et al. showed that for many frequently performed orthopedic procedures, evidence of causal superior effects of surgery compared with non-surgery has not yet been demonstrated [4]. An advantage of target trial emulation using real-world data is the ability to estimate causal effects in observational data, collected within standard care and including a more diverse range of participants. This makes the results more generalizable to the everyday patient population. Moreover, it can do so with reduced investments required in terms of time and money compared with performing an RCT [9].

### Limitations

In our study, differences in physical function, pain, and the patient’s global assessment of the target joint were assessed between the 2 groups. However, data on quality of life and adverse events, including mortality, was not collected. This represents a limitation, as it prevents a comprehensive assessment of all core health domains relevant to evaluating the effects of THA [10].

Additionally, given the constraints inherent in target trial emulation, our findings suggest a potential causal effect, but they do not provide the same level of evidence as a randomized controlled trial would. Not all characteristics were evenly distributed between the 2 groups, potentially indicating a confounding bias. In our study, ASA and BMI scores were not available for all patients at intake and other unmeasured confounding such as patients’ fear of being infected with COVID-19, socioeconomic status, or multi-morbidity may exist. The effect sizes with respect to hip function improvement and pain relief have been adjusted for confounding, yet our conclusions concerning causality must be approached with an appropriate degree of caution.

Another potential limitation of this study is the control group, which consists of patients on a waiting list. Literature suggests that patients awaiting an intervention might report less improvement than expected in a conservative cohort [17]. This “patient expectation bias” is especially the case for patients who were scheduled for surgery, but experienced short-notice cancellations. However, to a lesser extent, patient expectation bias is also expected to be present in an RCT, given the widely recognized clinical benefits of THA. Furthermore, the follow-up questionnaire for the non-surgery group was administered only temporarily during the COVID-19 pandemic, leading to missing data for patients in the control group and potential confounding. However, the sensitivity analysis performed on the imputed dataset showed similar, slightly stronger effects in favor of THA. We assumed data is missing at random, as questionnaire distribution stopped entirely without selective omission of specific patient groups. Patients were on the waiting list before planned surgery and follow-up questionnaires for the control group were sent in batches, resulting in a variability in follow-up time from intake, with durations extending beyond those in the THA group. Nevertheless, studies among patients with hip osteoarthritis, including control and placebo groups, indicate that patient-reported outcomes related to pain and hip function are anticipated to remain relatively stable over time [18,19].

### Conclusion

This study shows a substantial and clinically relevant effect of THA on hip disability compared with non-surgery in patients with hip osteoarthritis by means of target trial emulation.

*In perspective,* this study contributes to the evidence on the effect of THA compared with non-surgery in patients with hip osteoarthritis. A reporting guideline specifically for target trials aiming to emulate randomized controlled trials would improve the comparison between outcomes and applied methodology used in different study designs.

## Notes

Conception and design: RP, WJ, NW, AK. Analysis and interpretation of the data: RP, WJ, AK, RG. Drafting of article: AK. Critical revision of the article was performed by all authors. AK revised and submitted the manuscript.

The authors would like to thank data analysts Tryntsje Fokkema and Erik Til, and researcher Astrid de Vries from Martini Hospital for their help in this study. Additionally, thanks are offered to epidemiologist Amy Hofman from OLVG for her contribution regarding the additional sensitivity analyses.

Handling co-editors: Marianne Westberg and Robin Christensen

*Acta* thanks Johan Kärrholm and other anonymous reviewers for help with peer review of this manuscript.
